# The Sussex Oxford Compassion for the Self Scale validity in a working sample using classical test theory, item response theory and network analysis

**DOI:** 10.3389/fpsyg.2023.1110076

**Published:** 2023-05-12

**Authors:** Hongxing Meng, Xiaozhuang Wang, Hongpei Liu

**Affiliations:** ^1^Faculty of Psychology, Tianjin Normal University, Tianjin, China; ^2^Academy of Psychology and Behavior, Tianjin Normal University, Tianjin, China; ^3^Tianjin Social Science Laboratory of Students’ Mental Development and Learning, Tianjin Normal University, Tianjin, China

**Keywords:** self-compassion, Sussex Oxford Compassion for the Self Scale, item response theory, network analysis, Chinese occupational groups

## Abstract

Self-compassion is a relatively new construct in the scientific literature, and there is currently a lack of robust psychometric measures of self-compassion in the workplace. Therefore, validating the Sussex Oxford Compassion for the Self Scale (SOCS-S) in various cultural settings is essential to add to the existing research on the psychometric properties of the scale. This study aimed to evaluate the validity of the SOCS-S in a Chinese working sample of 1,132 participants (39.4% males) using classical test theory (CTT), item response theory (IRT), and Network Analysis. The results supported the validity of the SOCS-S’s five-factor structure, with high internal consistency and measurement invariance across genders. IRT was applied using a graded response model (GRM) to assess the overall SOCS-S scale items, indicating that all 20 items had sufficient discrimination indices and acceptable difficulty indices. Moreover, it is worth noting that the results of the network analysis are consistent with those of the IRT analysis. In summary, the study confirms the validity of the SOCS-S as a scale for assessing self-compassion among Chinese occupational groups.

## Introduction

Previous research has confirmed that self-compassion plays a positive role in helping people cope with the frustrations and pains of daily life (Ewert et al., 2021) and is also important in organizational life (Janssen et al., 2018). Self-compassion is a fundamental concept in positive psychology, which is grounded in Buddhist psychology’s notion of “compassion.” The construct of self-compassion (SC) was introduced, conceptualized, and operationalized by Kristin D. Neff. Self-compassion comprises three bi-polar and mutually interacting dimensions: self-kindness versus self-judgment; common humanity versus isolation; and mindfulness versus over-identification (Neff, 2003). Compassion for oneself is considered a healthy, positive self-concept. In recent years, researchers have found that self-compassion can effectively enhance an individual’s intrinsic motivation, reduce negative emotions and improve the ability to adjust positively to adverse events (Stutts et al., 2018; Andersson et al., 2021; Hughes et al., 2021). Self-compassion is associated with various positive psychological outcomes, including greater mental resilience (Kotera et al., 2021a,b), higher levels of well-being (Passmore et al., 2018), and the ability to enhance individual levels of self-compassion through appropriate interventions (Wilson et al., 2019).

Self-compassion also plays a vital role in organizational life, where suffering is an unavoidable part (Dutton et al., 2014). At work, employees frequently experience unpleasant feelings and sensations due to various workplace occurrences, such as workplace, including workplace restrictions (Yu et al., 2021), toxic interactions with coworkers (Quade et al., 2019), and failed entrepreneurial endeavors (Patzelt et al., 2021). It is also possible for pain to arise from unrelated events and spread into the workplace (Eby et al., 2016), such as employment-related difficulties (Oka et al., 2017), past traumatic experiences (Izutsu et al., 2008), and global pandemics (Guler et al., 2021). Notably, emerging organizational studies have started to investigate how practicing self-compassion in the face of adversity can positively affect organizational outcomes. Researchers have documented that individuals high in self-compassion can effectively handle difficult situations and cope better with burnout (Terry et al., 2013). Meanwhile, researchers have also found that self-compassion could buffer the negative relationship between work–family conflict and psychological well-being (Rafique et al., 2018; Yang et al., 2022).

Countries have recently conducted research on self-compassion in the workplace, exploring its role in facilitating and protecting employees’ responses to setbacks and challenges. For example, a study based on a European professional group found that mindfulness training increased employees’ self-compassion, which in turn reduced their stress and fatigue (van der Meulen et al., 2021); a cross-sectional study in Japan found that employee mental health problems were significantly and negatively associated with levels of self-compassion (Kotera et al., 2022); a study based on a U.S. occupational group found that employees with a self-compassionate mindset were better able to cope with feelings of job isolation and depression (Andel et al., 2021). Although evidence of the positive role of self-compassion in the workplace has been obtained from studies in both eastern and western cultural contexts, however, research in Chinese occupational groups is underrepresented. This is reflected in the inadequacy of both the research instrument and the research findings. As the Eastern country with the most significant number of labor resources, exploring the manifestation and influence mechanism of self-compassion in Chinese occupational groups is undoubtedly indispensable to enrich the empirical research results of self-compassion and deepen the theoretical understanding of self-compassion in the Eastern cultural context.

Valid and reliable instruments are used to measure self-compassion in research. The Self-Compassion Scale (SCS; Neff, 2003) and its short-form variant (SCS-SF; Raes et al., 2011) are the most commonly used scales measuring six different aspects of self-compassion, which includes self-kindness, self-judgment, common humanity, isolation, mindfulness, and over-identification. Nevertheless, some researchers argue that empirical studies do not always support the six-factor model. Instead, they suggest that the SCS and SCS-SF only represent two aspects: self-criticism and self-compassion (Muris et al., 2016; Babenko and Guo, 2019; Halamová et al., 2021). To address this, Strauss et al. (2016) integrated different definitions and measures of self-compassion from existing research, suggesting that self-compassion is a cognitive, emotional, and behavioral dynamic process that involves (1) recognizing suffering, (2) understanding that suffering is a part of every human experience, (3) feeling empathy and relating to people who are suffering, (4) tolerating unpleasant feelings caused by the sufferer, and (5) taking action to ease discomfort. Based on the five-factor structure described above, Gu et al. (2020) developed the Sussex-Oxford Compassion for the Self (SOCS-S) and the Sussex-Oxford Compassion for Others Scale (SOCS-O). Both scales consist of five elements that constitute overall self-compassion and show good reliability and validity with their original samples.

Based on the above theoretical conceptualization and scale development, the SOCS-S has been shown to be a reliable and valid tool for measuring self-compassion in various countries and industries (Kim and Seo, 2021; de Krijger et al., 2022; Lucarini et al., 2022; Shreffler et al., 2022). However, to further validate its use, it is necessary to examine its measurement invariance across gender and culture and to measure self-compassion levels in different occupational groups in various countries. To the best of our knowledge, there is no validation study of SOCS-S in Chinese to date.

Therefore, the purpose of this study is twofold. First, we aim to develop a Chinese version of the SOCS-S and evaluate its psychometric properties by administering it to a Chinese occupational group. We used classical test theory (CTT), which includes internal consistency, factor analysis, and measurement invariance across gender. Internal consistency assessed the reliability of the SOCS-S scale without retesting. Factor analysis examined whether the same factor structure would be found in a Chinese sample. Measurement invariance cross-validated the five-factor model across gender groups. We also utilized modern approaches such as item response theory (IRT), also known as graded response model analysis (GRM), and SOCS-S network analysis. Self-compassion at work refers to the extent to which employed individuals are well prepared (cognitively and emotionally) to effectively handle the difficulties faced in their organizations while on the job. We are interested in understanding how much self-compassion Chinese employees require to respond using each scale category, and how effectively each item differentiates (the α parameter) between employees with varying degrees of self-compassion (the *b* parameters). Furthermore, we conducted a network analysis to examine the strength, proximity, and betweenness indices of every node included in the estimated network. The traditional approach to psychometric measurement of scales is to conduct factor analysis, mainly focusing on the differences in the included items (Sellbom and Tellegen, 2019). However, this traditional method only calculates the correlation of the included items. Network analysis presents the characteristics and information of a scale in the form of a network consisting of nodes and edges, to visualize the structure and information of a scale by means of the recurrence of these relations, and to describe and explain the scale from the perspective of the network (Contreras et al., 2019). Simultaneously, it confirms the feasibility of SOCS-S as a measurement tool in Chinese occupational groups. By measuring the level of self-compassion in Chinese occupational groups and exploring the adaptability of the SOCS-S in different cultural contexts, we provide instrumental support for further research related to self-compassion in the workplace in China.

## Methods

### Participants and procedure

The research recruited 1,132 working participants, comprising 446 males (39.4%) and 686 females (60.6%), using a convenience sampling method. We utilized social networking sites and the WeChat application to distribute the online survey and ensure that each participant could only submit one form. Informed consent was obtained from all participants, and they were informed of the study’s purpose on the first page of the survey. Initially, 1,302 working participants were enrolled, but those with too short response times were excluded, resulting in a 1.3% attrition rate. The final sample size for this study was 1,132.

The current study was conducted voluntarily, and the responses provided on the questionnaires were kept anonymous and confidential by the requirements of the protocol governing the collection of data. The authors affirm that the research subjects were treated by APA ethical standards and claim no conflicts of interest. Table 1 provides a descriptive statistical analysis of the participants’ sociodemographic characteristics.

**Table 1:** Demographic characteristics of the participates in the validation sample (*N* = 1,132).

		Validation sample N (%)
Gender	Male	446 (39.4)
	Female	686 (60.6)
Age	20 years and below	91(8)
	21–30	618 (54.6)
	31–39	308 (27.2)
	40–49	91 (8.0)
	50 years and above	24 (2.1)
Education	High school or below	197 (17.4)
	Post-secondary	280 (24.7)
	Bachelor	539 (47.6)
	Master or above	116 (10.2)
Occupation	Production staff	77 (6.8)
	Sales staff	107 (9.5)
	Marketing staff	52 (4.6)
	Technical staff	144 (12.7)
	Management	132 (11.7)
	Faculty	104 (9.2)
	Professionals	145 (12.8)
	Others	371 (32.8)

## Measures

### Sociodemographic variables

To provide an overview of the participants’ sociodemographic characteristics, the survey included questions regarding gender, age, educational level, and occupation.

### Sussex-Oxford Compassion for the Self Scale

The SOCS-S is a 20-item questionnaire designed to assess levels of self-compassion. The scale was developed based on Strauss et al. (2016) five core characteristics of self-compassion: recognizing suffering, understanding the universality of suffering, feeling empathy for the person suffering, tolerating uncomfortable feelings, and taking action to alleviate suffering. Participants rated their responses on a 5-point Likert scale, ranging from 1 (*not at all true*) to 5 (*always true*). A higher score indicates a greater level of self-compassion. The present study obtained a Cronbach’s alpha value of 0.92 (95% CI [0.92, 0.93]), indicating high internal consistency. According to the preliminary research conducted by Gu et al. (2020), the value of Cronbach’s alpha ranged from 0.75 to 0.93. The forward-backward translation approach was used to translate the SOCS-S into Chinese. After the authors validated the initial translation, a bilingual psychology student who had lived in an English-speaking country for more than 15 years back-translated the questionnaire (see Supplementary material).

### Analysis strategy

For data recording, we used the software R (version 4.2.0) packages psych (Revelle, 2022), lavaan (Rosseel, 2012), mirt (Chalmers, 2012), and qgraph (Epskamp et al., 2012) were used. The procedure is as follows: (1) Confirmatory factor analysis (CFA) was used to examine the reliability of the measures used to assess the concept validity, and four fit indices were used to indicate model fit: root mean square error of approximation (RMSEA; Steiger, 1990), the standardized root means square residual (SRMR), comparative fit index (CFI; Bentler, 1990) and the Tucker-Lewis index (TLI; Tucker and Lewis, 1973). The CFI and TLI should be close to or greater than 0.90, and both RMSEA and SRMR should be lower than or equal to 0.08 are considered satisfactory (Hu and Bentler, 1999); (2) We estimated the internal consistency of the scale and each dimension; (3) A multi-group CFA analysis was carried out across genders to evaluate the measurement invariance of the best-fitting model. Invariance degree was evaluated by including limitations (configural invariance model, metric model, scalar model), as well as by assessing the changes that occurred between models in χ^2^, in the CFI, and RMSEA values (Asparouhov and Muthén, 2014; Brown, 2015); (4) Before applying an IRT model to a scale, the assumption of unidimensionality must be evaluated. However, according to the findings of Reckase (1979), it is sufficient to focus on a single dominant factor that significantly impacts the item answer to proceed with the study. Indicators supporting the assumption of unidimensionality include: (a) the first factor accounts for at least 20% of the variance (Reckase, 1979); or (b) the ratio of the eigenvalues of the first and second factors is greater than 3 (Morizot et al., 2007); or a conformity to the unidimensional model that is acceptable according to the confirmatory factor analysis (CFA); (5) We applied the 2PLM IRT analyses and selected a graded response model (GRM) following the SOCS-S five response categories (1–5),. The GRM estimates the standard item discrimination parameter (α) as well as the difficulty threshold parameter (*b*) position for each answer category inside the item. According to Baker (2001), items with a discrimination score exceeding 1.7 are considered to be very informative. Scale items with values outside the range − 3 to +3 may be problematic due to improper wording, or less usable answer categories; (6) We used item correlation research as the foundation for a network analysis of the SOCS-S, which was used to investigate the scale’s underlying structure as a visual representation. Three node centrality indices (strength, closeness, and betweenness) were calculated to provide quantitative descriptions of the structural importance of each node in the network. Estimated network nodes that are more centrally located exhibit higher centrality values (Epskamp et al., 2018; Dodson and Heng, 2021).

## Results

### SOCS-S validity using CTT (classical test theory)

#### Factor structure of The SOCS-S

In terms of the one-factor model, the chi-square test showed a significant result. However, the generated fit indices indicated poor model fit [χ^2^(170) = 1687.363, *p* < 0.001; RMSEA = 0.089; SRMR = 0.062; CFI = 0.853; TLI = 0.836]. Therefore, based on the comparison of the five-factor models, most fit indices suggested that the five-factor structural model was a better fit, and all item loadings were statistically significant (Table 2). The fit indices and factor loadings indicated that the five-factor model of the SOCS-S provided the best fit to the data [χ^2^(160) = 846.954, *p* < 0.001; RMSEA = 0.062; SRMR = 0.039; CFI = 0.934; TLI = 0.921].

**Table 2:** Fit indices for SOCS-S models tested.

Model	CFI	TLI	RMSEA (90%CI)	SRMR	χ^2^
One-factor model	0.853	0.836	0.089 (0.085, 0.096)	0.062	1687.363 (170)
Five-factor model	0.934	0.921	0.062 (0.058, 0.066)	0.039	846.954 (160)

CFI, Comparative Fit Index; TLI, Tucker-Lewis index; RMSEA, Root Mean Square Error of Approximation; CI, Confidence Interval; SRMR, Standardized Root Mean Square Residuals; χ^2^, chi-square; df, degree of freedom.

#### Internal consistency

Reliability analysis is a crucial step in assessing the quality of psychometric measures. However, traditional measures of reliability, such as Cronbach’s α, can be problematic when applied to ordinal scales (Zumbo et al., 2007; Dunn et al., 2014; McNeish, 2018). To address this issue, researchers have proposed alternative measures, including McDonald’s omega (Dunn et al., 2014) and the IRT reliability coefficient (Kim and Feldt, 2010), which are better suited to ordinal scales. In this study, we used McDonald’s omega to assess the reliability of the SOCS-S, but we also reported Cronbach’s alpha for comparison. The values for Cronbach’s alpha for the total SOCS-S and its subscales ranged from 0.706 to 0.927, while McDonald’s omega estimates for the total SOCS-S and its subscales ranged from 0.819 to 0.936. These results suggest that the SOCS-S has good internal consistency. The inter-item correlations ranged from 0.504 to 0.899 and were all positive, indicating that the items are measuring the same underlying construct (see Supplementary material). The item-total correlations ranged from 0.754 to 0.888 and were all positive, indicating that each item is contributing to the overall reliability of the measure. Depending on Kline’s (2013) criteria, these values were deemed adequate for measuring psychological constructs. Table 3 provides detailed information on the McDonald’s ω and Cronbach’s α values for both the entire SOCS-S scale and its individual subscales.

**Table 3:** Reliability of SOCS-S scale and subscale items.

Composite scale score	Item	McDonald’s ω	Cronbach’s α	Item-total correlation	α if item deleted
Recognizing suffering	1, 6, 11, 16	0.844	0.750	0.779	0.933
Understanding the universality of suffering	2, 7, 12, 17	0.819	0.706	0.754	0.935
Feeling for the person suffering	3, 8, 13, 18	0.871	0.803	0.899	0.912
Tolerating uncomfortable feelings	4, 9, 14, 19	0.847	0.756	0.878	0.918
Acting or being motivated to act to alleviate suffering	5, 10, 15, 20	0.880	0.817	0.888	0.914
Total scale		0.936	0.927	–	0.897

*N* = 1,132.

#### Measurement invariance testing

Measurement invariance testing was conducted to examine the measurement invariance between women and men (see Table 4). Configural invariance models of the SOCS-S were tested for women and men. The results indicated a good fit, suggesting the equivalent factor structure of SOCS-S for both gender groups [χ^2^_diff_(30) = 36.47, *p* < 0.001; ΔRMSEA = 0.003]. Metric invariance constrained the factor loadings to be equivalent across gender groups while allowing item intercepts to vary freely. The results supported equivalent factor loadings, which suggested that the five factors of the SOCS-S were assessed by respective items in a similar manner across the gender groups [χ^2^_diff_(15) = 14.99, *p* < 0.001; ΔRMSEA = 0.001]. Scalar invariance was conducted to examine whether the item intercepts were equivalent for people of different genders. The analyses supported the intercept equivalence in SOCS-S, suggesting that one or more parameters were equivalent across gender groups [χ^2^_diff_(15) = 21.48, *p* < 0.001; ΔRMSEA = 0.002]. Relevant changes in CFI values (ΔCFI) ≤ 0.01, as well as changes in RMSEA values (ΔRMSEA) ≤ 0.015, were evidence of adequate measurement invariance (Cheung and Rensvold, 2002). It has been demonstrated that the configural, metric, and scalar invariance models have all successfully attained measurement invariance across gender.

**Table 4:** Analysis of measurement invariance for the SOCS-S across gender.

Models	χ^2^(*df*)^a^	CFI^b^	RMSEA^c^	Δχ^2^	ΔCFI	ΔRMSEA
General	1145.690 (350)	0.924	0.063	–	–	–
Configurational	1109.220 (320)	0.924	0.066	36.470(30)^***^	0	0.003
Metric	1124.210 (335)	0.924	0.065	14.990(15)^***^	0	0.001
Scalar	1145.690 (350)	0.924	0.063	21.480(15)^***^	0	0.002

^a^χ^2^, chi-squared; df, degrees of freedom; ^b^CFI, Comparative Fit Index; ^c^RMSEA, Root Mean Square Error Approximation; Δ, Delta. ^***^p < 0.001.

### SOCS-S validity using IRT

#### Unidimensionality check

Table 5 displays the descriptive statistics of the Chinese version of the SOCS-S, including the total scale score and each of the five domain-specific subscale scores which were generated based on the existing literature (Gu et al., 2020). Though CFA with maximum-likelihood estimation did not support data unidimensionality [χ^2^(170) = 1687.363; RMSEA = 0.089; SRMR = 0.062; CFI = 0.853; TLI = 0.836], the SOCS-S met the strict assumption of unidimensionality (Morizot et al., 2007). The first-to-second eigenvalue ratio was more significant than three, indicating a preponderant unidimensionality component that explained at least 20% of the variance for the entire questionnaire and each of its five domains (see Table 5). These results were deemed to meet the standards for “good enough” unidimensionality (Cho et al., 2015).

**Table 5:** Descriptive statistics of the Chinese version of the SOCS-S.

Composite scale score	Mean	*SD*	Skewness	Kurtosis	% variance 1st factor; 2nd factor	First/Second eigenvalue
Recognizing suffering	3.818	0.715	−0.294	0.026	57.610; 16.200	2.310/0.640 = 3.610
Understanding the universality of suffering	4.085	0.674	−0.714	0.748	53.140; 17.580	2.120/0.700 = 3.020
Feeling for the person suffering	3.877	0.761	−0.481	0.237	62.870; 14.310	2.510/0.570 = 4.400
Tolerating uncomfortable feelings	3.635	0.799	−0.27	−0.140	58.190; 17.170	2.320/0.680 = 3.410
Acting or being motivated to act to alleviate suffering	3.921	0.765	−0.517	0.038	64.720; 13.850	2.580/0.550 = 4.690
Total scale	3.867	0.626	−0.289	0.306	42.520; 8.610	8.500/1.720 = 4.940

*N* = 1,132.

#### Item parameter estimates for the SOCS-S

The results, as shown in Table 6, indicate that the discriminating parameter (α) had values ranging from 1.01 to 3.15. According to Baker (2001) criteria, a discriminating parameter index of less than 0.64 indicates unacceptable discrimination, between 0.65 and 1.34 indicates moderate discrimination, between 1.35 and 1.69 indicates high discrimination, and more than 1.70 indicates extremely high discrimination. Thus, our findings demonstrate that all items of the SOCS-S not only made a significant contribution but also had a good capacity to discriminate the underlying self-compassion dimension.

**Table 6:** Item parameters—slopes and thresholds for the SOCS-S items.

Item	SOCS-S domain	α	B1	B2	B3	B4
SOCS-S1	RS	1.18	−4.51	−3.03	−1.12	0.90
SOCS-S2	US	1.01	−5.18	−3.61	−1.65	0.13
SOCS-S3	FS	2.11	−2.86	−1.73	−0.51	0.23
SOCS-S4	TF	2.03	−2.36	−1.35	−0.35	0.78
SOCS-S5	AM	2.01	−2.73	−1.78	−0.62	0.64
SOCS-S6	RS	1.30	−4.01	−2.54	−1.07	0.73
SOCS-S7	US	1.28	−3.78	−2.56	−1.29	0.23
SOCS-S8	FS	2.24	−2.59	−1.62	−0.56	0.63
SOCS-S9	TF	2.41	−2.50	−1.46	−0.42	0.65
SOCS-S10	AM	2.88	−2.61	−1.62	−0.63	0.42
SOCS-S11	RS	1.45	−2.59	−1.35	−0.05	1.25
SOCS-S12	US	1.41	−3.27	−2.21	−0.89	0.58
SOCS-S13	FS	2.43	−2.68	−1.81	−0.79	0.43
SOCS-S14	TF	1.99	−2.33	−1.42	−0.23	0.99
SOCS-S15	AM	2.26	−2.59	−1.72	−0.65	0.55
SOCS-S16	RS	1.76	−3.06	−1.93	−0.62	0.88
SOCS-S17	US	1.36	−3.63	−2.49	−1.01	0.63
SOCS-S18	FS	2.86	−2.34	−1.46	−0.44	0.62
SOCS-S19	TF	1.52	−2.53	−1.42	−0.25	1.11
SOCS-S20	AM	3.15	−2.38	−1.52	−0.61	0.40

α, discrimination parameter; B1-B4, difficulty parameter.

Regarding discrimination values, item 2 (i.e., “I understand that everyone experiences suffering at some point in their lives”), item 7 (i.e., “I understand that feeling upset at times is part of human nature”), item 12 (i.e., “Like me, I know that other people also experience struggles in life”), and item 17 (i.e., “I know that we can all feel distressed when things do not go well in our lives”), which were belong to the US domain (*Understanding the universality of suffering*), had lower values than all other items. These values were above the cut-off (> 0.6), indicating a moderate capacity to distinguish between latent constructs with low and high levels. On the other hand, item 5 (i.e., “I try to make myself feel better when I’m distressed, even if I cannot do anything about the cause”), item 10 (i.e., “When I’m going through a difficult time, I try to look after myself”), item 15 (i.e., “When I’m upset, I try to do what’s best for myself”), and item 20 (i.e., “When I’m upset, I do my best to take care of myself”), which were belong to the AM domain (*Acting or being motivated to act to alleviate suffering*), had higher values ranging from 2.01 to 3.15. The values of the remaining items, which ranged from 1.18 to 2.86, were also high. In summary, these findings suggested that the items measuring various self-compassion domains operationalized by the SOCS-S scale had a proper balance regarding discriminative power.

To estimate each item’s difficulty, the difficulty parameter (*b*) was used (Muraki, 1992). Positive (*b*) values imply more incredible difficulty, negative (*b*) values suggest a greater difficulty, and values close to zero indicate moderate difficulty (Reise and Henson, 2003). The values of the four location parameters (*b*1, *b*2, *b*3, and *b*4) were very similar for all items. All items had a high and negative value for parameter *b*1 (ranging from −5.18 to −2.34), while parameter *b*2 had a negative value for all items but lower than *b*1 (ranging from −3.61 to −1.35), parameter *b*3 also had a negative value for all items but lower than *b*2 (ranging from −1.65 to −0.23), and *b*4 had a positive value (ranging from 0.13 to 1.25). Overall, these findings suggest that respondents with high self-compassion tend to select *always true* for the items, while those with low self-compassion tend to select *not at all true*.

#### Item information function

**Figure 1:**
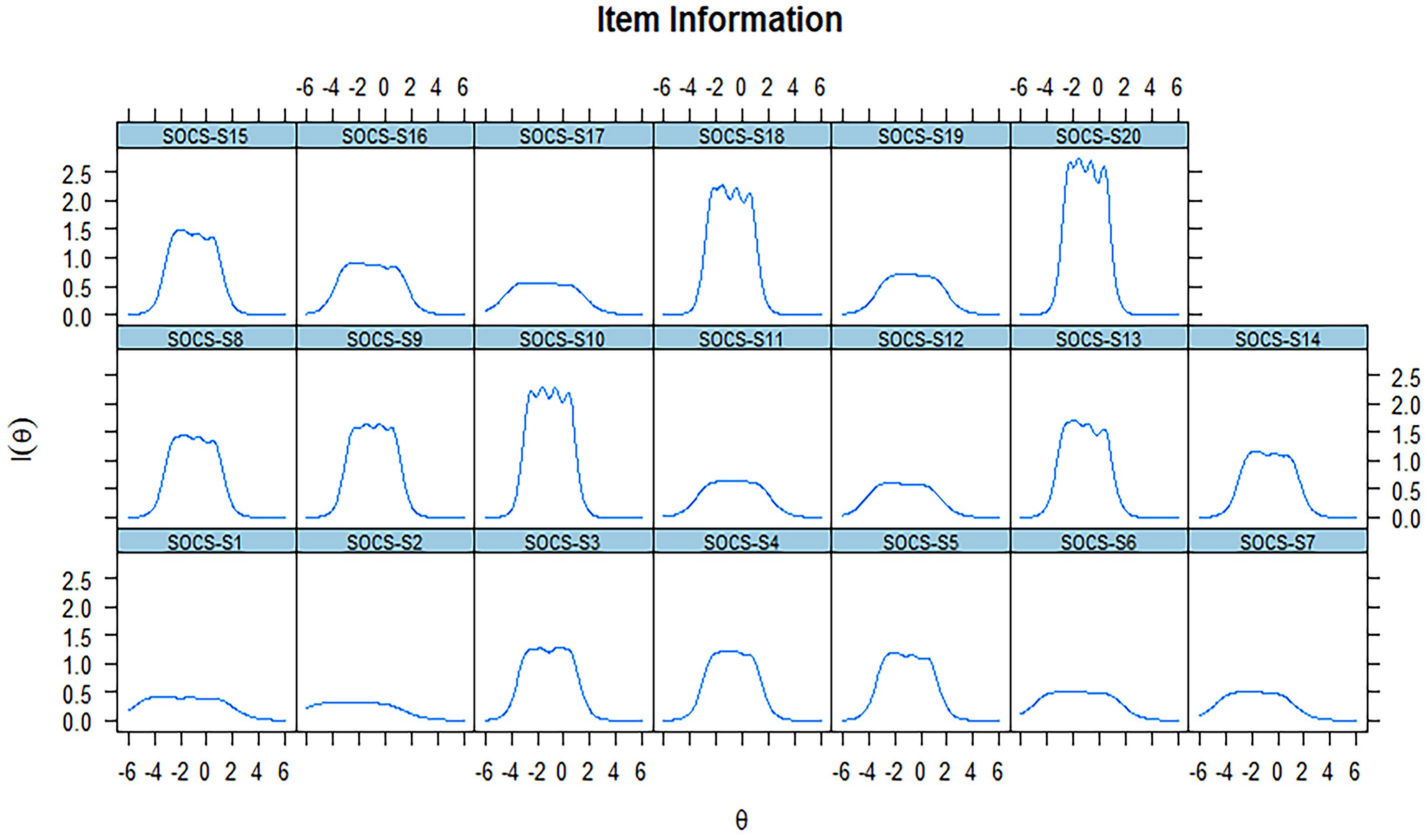
Item information function for the SOCS-S Scale (20 items).

**Figure 2:**
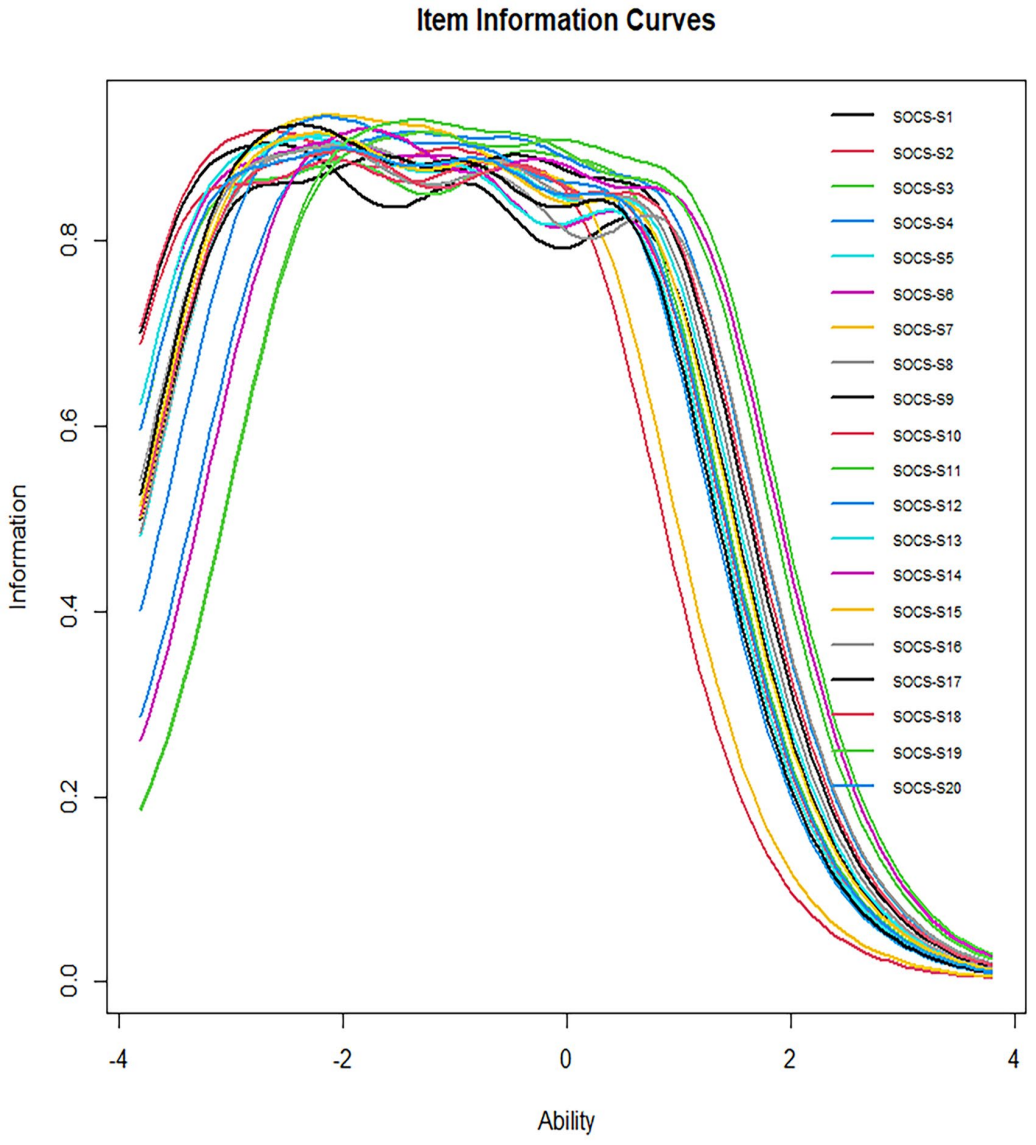
Item information curves (IICs) for each item of the SOCS-S.

The study presented item information functions (IIFs) and item information curves (IICs; Figures 1, 2), which showed that the IIFs were unimodal (Item 4, 11, 12, 19), bimodal (Item 3, 5, 14, 16) and multimodal (Item 8, 9, 10, 13, 15, 18, 20). This is not surprising, as each response category (ranging from 1 to 5) contributes its information, which may peak within different attribute ranges (Stanculescu, 2022).

Three observations can be made about the IIFs and IICs: (1) Eight items (1, 2, 6, 7, 11, 12, 17, 19) provided less information than others, but their amount of information provided was ranked similarly. These items accurately measured one’s level of self-compassion, ranging from low to high. (2) Twelve items (3, 4, 5, 8, 9, 10, 13, 14, 15, 16, 18, 20) provided the most information for people with self-compassion around θ = −1. (3) Six items (1, 2, 6, 7, 12, 17) were too “easy,” suggesting higher response categories that required too little latent self-compassion. Moreover, they provided virtually no information to individuals whose scores were more than three standard deviations above the θ mean. Item characteristic curves were also included in the Supplementary material.

#### Test information function

**Figure 3:**
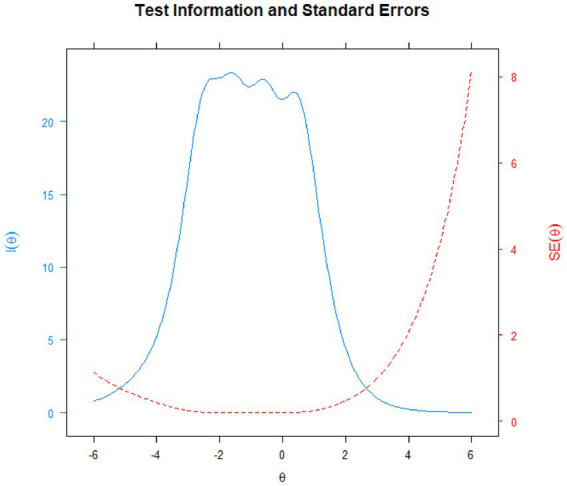
Test information function (blue line) and standard errors (red line) for the SOCS-S.

**Figure 4:**
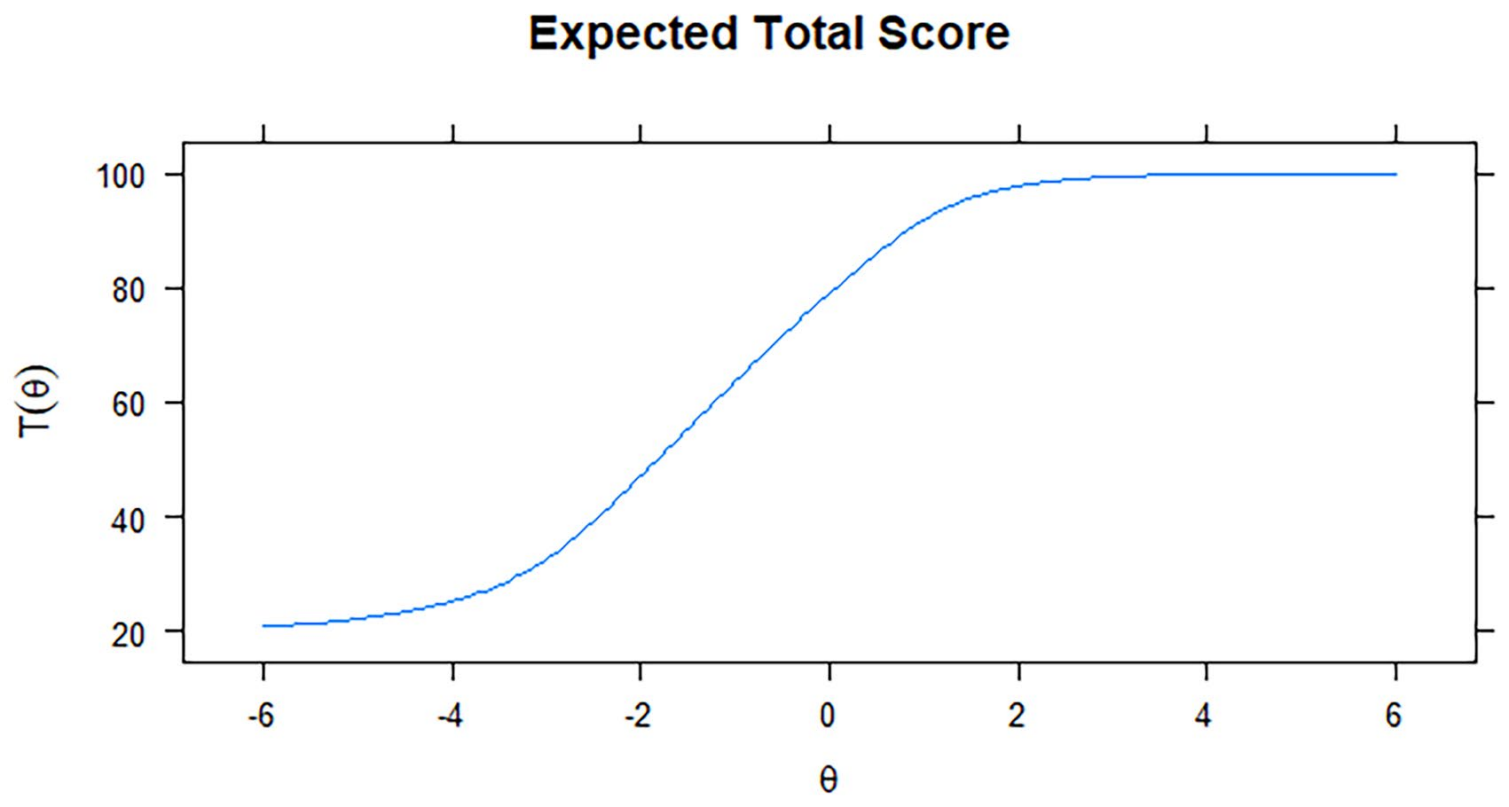
Test characteristic curve for the SOCS-S.

Figures 3, 4 depict the Test Information Function (TIF), which provides information about the reliability of the SOCS-S. The test information function is shown by the blue line, which displays the accuracy degree of the difficult values, how much information the items provide, and the proportion of the scale that covers the score range. For the SOCS-S, the TIF provides relatively consistent information about individuals between [−3; 1], with a decline in either direction beyond these points. The scale provides a significant quantity of information between −0.5 and + 2.5 *SD* from the mean, with a peak around -1*SD*.

The red line depicts the conditional standard errors, indicating how precision estimation changes over θ, with smaller values showing higher precision estimation. Since the conditional standard errors (*SE*s) reflect the scale information function, the optimal range for estimated score accuracy was −3 < θ < +1. Although *SE* values suggest that scale precision was best within the −3 < θ < +1 range, scores outside this range were not useless. The average for *SE*s across score patterns was 0.24, with a standard deviation of 0.08, and the range of *SE*s was 0.17–0.42. Brown and Croudace (2015) suggested a threshold value of 0.5 to assess the adequacy of the precision of θ scores (p. 16). Less than 1% of our *SE*s were higher than 0.5, predominantly located in the upper range of θ. Therefore, the TIF shows that the SOCS-S performs well, providing accurate estimations of scores throughout a broad spectrum of the continuum, with acceptable marginal reliability and low *SE*.

#### Network analysis of the SOCS-S

**Figure 5:**
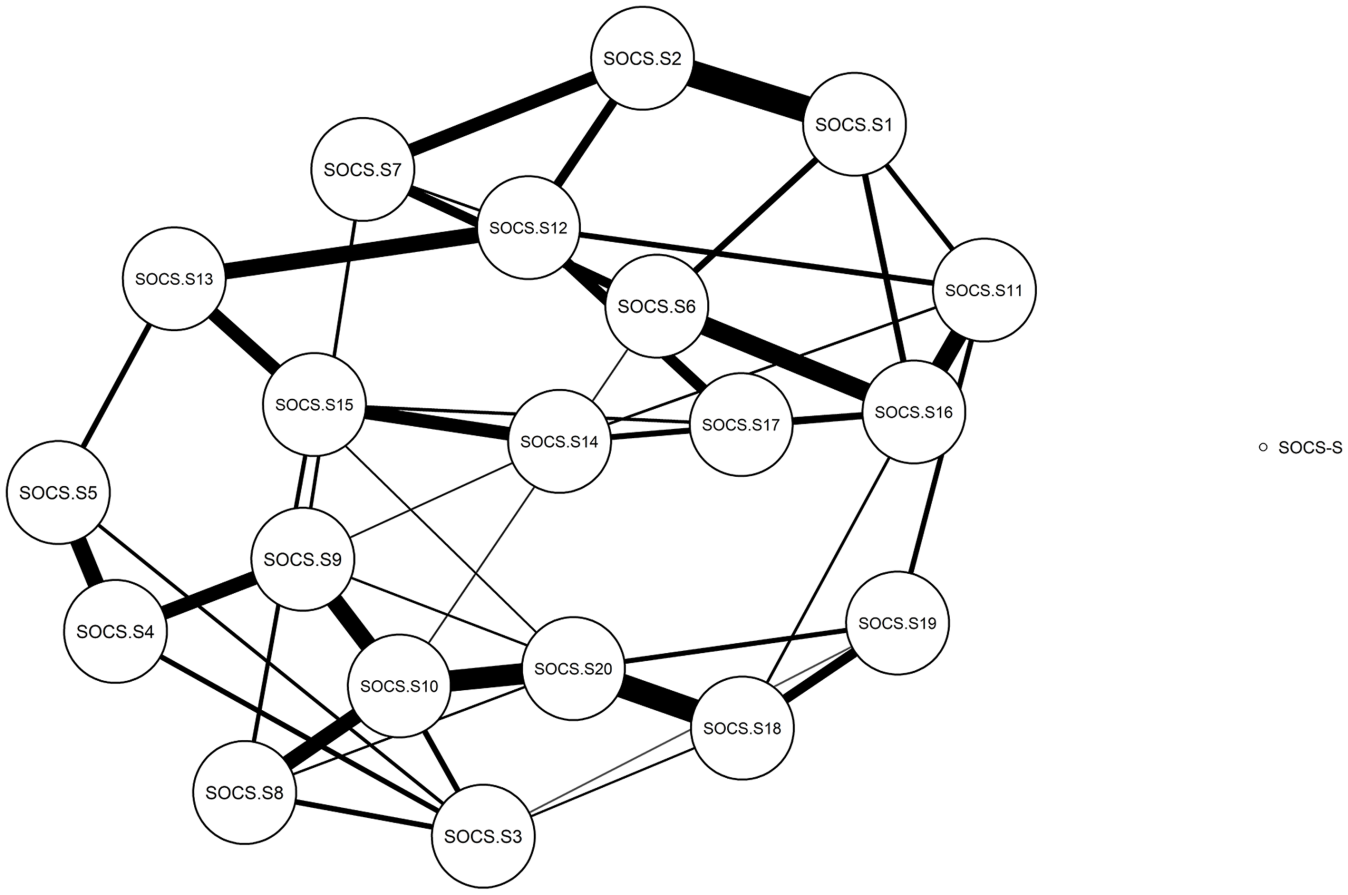
Estimated correlation network of the SOCS-S (20 items).

**Figure 6:**
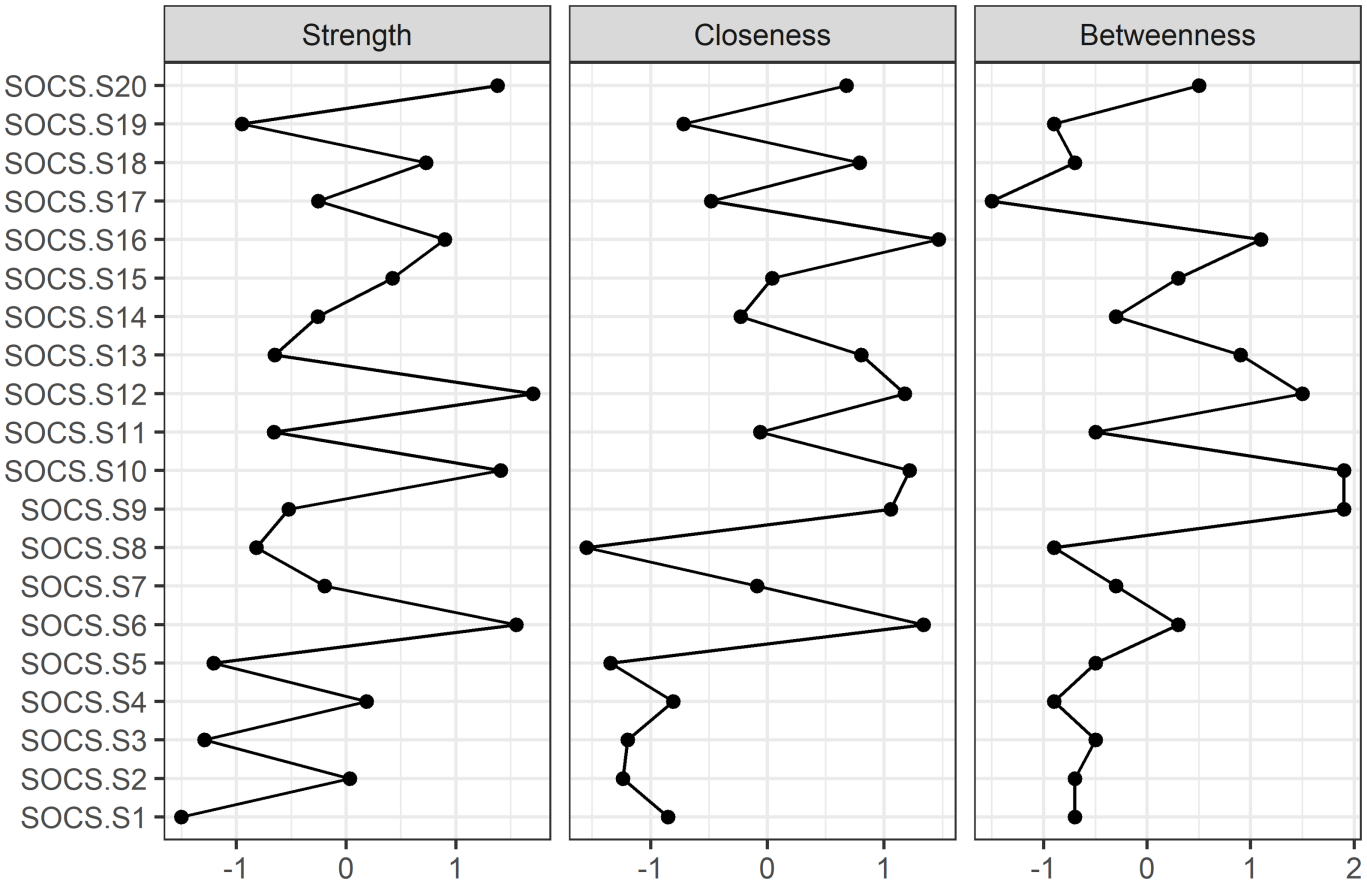
Betweenness, closeness, and node strength centrality estimates for the SOCS-S (Z-scores).

Upon visual inspection of the estimated network (see Figures 5, 6), several features are notable. First, specific nodes, such as item 20 (i.e., “When I’m upset, I do my best to take care of myself”) and item 12 (i.e., “Like me, I know that other people also experience struggles in life”) and item 6 (i.e., “I notice when I’m feeling distressed”), exhibit strong connectivity with the rest of the network. In contrast, others appeared to be on the periphery, such as item 3 (i.e., “When I’m going through a difficult time, I feel kindly toward myself”) and item 8 (i.e., “When bad things happen to me, I feel caring toward myself”).

Second, the local network structure investigation indicated that four nodes were more central than the rest, as they had the greatest centrality index, namely item 20 (i.e., “When I’m upset, I do my best to take care of myself”) (strength index = 1.38), item 12 (i.e., “Like me, I know that other people also experience struggles in life”) (strength index = 1.70), item 10 (i.e., “When I’m going through a difficult time, I try to look after myself”) (strength index = 1.41) and item 6 (i.e., “I notice when I’m feeling distressed”) (strength index = 1.55). Moreover, item 1 (i.e., “I’m good at recognizing when I’m feeling distressed”) had the weakest direct connection (strength index = −1.50).

Third, in terms of the closeness index, items 16 (i.e., “I recognize signs of suffering in myself”) (closeness index = 1.47) and 6 (i.e., “I notice when I’m feeling distressed”) (closeness index = 1.34) had the highest indirect connections, while item 8 (i.e., “When bad things happen to me, I feel caring toward myself”) (closeness index = −1.55) had the lowest.

Fourth, with regard to the betweenness index, item 9 (i.e., “I connect with my own distress without letting it overwhelm me”) (betweenness index = 1.90) and item 10 (i.e., “When I’m going through a difficult time, I try to look after myself”) (betweenness index = 1.90) had the highest scores, as they were frequently located on the shortest path between two other nodes in the network. In contrast, item 17 (i.e., “I know that we can all feel distressed when things do not go well in our lives”) was the least common on such paths (betweenness index = −1.50).

## Discussion

The purpose of this study was to assess the psychometric properties of the Sussex Oxford Compassion for the Self Scale (SOCS-S) in a Chinese occupational group. The SOCS-S was translated and administered to the Chinese occupational group to overcame the limitations of previous self-compassion scales, specifically its good content validity in ensuring that the items are relevant to suffering and not overlapping with other constructs. Thus, the scale helps us in understanding the level of self-compassion in the Chinese occupational group. Our findings support the factor structure of the SOCS-S and its robust psychometric properties.

Consistent with a previous study (Gu et al., 2020), this research found high internal consistency for the SOCS-S, as evidenced by McDonald’s ω (0.819–0.936) and Cronbach’s α (0.706–0.927). The five-factor structure showed reasonable model fit for the scale. Multi-group CFA analyses validated the measurement invariance across gender for the SOCS-S, indicating that males and females had similar interpretations of the item content. These findings are consistent with earlier observations (Gu et al., 2020). However, our results differ from Kim and Seo (2021) study, which failed to find scalar measure invariance. Other studies, such as de Krijger et al. (2022), only found metric invariance. This indicates the need for further research on the measurement invariance of the SOCS-S across gender, as the correlations between the five characteristics of self-directed compassion in Chinese working populations may differ from those in other cultures. One possible explanation for these cultural differences in self-compassion is that it is a social mindset that can be influenced by various social environments (Gilbert, 2014). In China, gender equality is an important part of education (Zhu et al., 2018). Since the founding of the People’s Republic of China, the reform of institutions, laws and policies has promoted the popularization of gender equality concepts and the de-gendering of social roles. Therefore, when facing pain and setbacks in work and life, both men and women have similar coping strategies. Furthermore, “home culture” is a unique cultural concept of Chinese enterprises, which originates from the profound understanding and importance of “home” among Chinese people (Huang et al., 2022). In the Chinese perspective, “home” not only refers to a family or a clan, but also encompasses the nation and the world. Therefore, enterprises should be united and harmonious like a big family, and develop together. “Persistence” and “self” are two important elements of home culture (Dang and Zhao, 2020). Only by pursuing goals persistently can the team improve its execution and efficiency; only by establishing correct self-awareness and values can the team maintain its personality and creativity.”

This study confirmed that the SOCS-S met a strict assumption of unidimensionality (Morizot et al., 2007) with: (1) a ratio greater than 3 between the first and second eigenvalues; and (2) a dominant factor explaining at least 20% of the variance. The GRM analysis revealed that the SOCS-S is a psychometrically sound instrument, as the slopes and thresholds provided informative indicators of individuals’ self-compassion levels. The ICC curves show that item 5 (i.e., “I try to make myself feel better when I’m distressed, even if I cannot do anything about the cause”), item 10 (i.e., “When I’m going through a difficult time, I try to look after myself”), item 15 (i.e., “When I’m upset, I try to do what’s best for myself”), and item 20 (i.e., “When I’m upset, I do my best to take care of myself”) exhibit higher discrimination and are more useful in assessing the latent trait related to taking action or being motivated to alleviate suffering. In contrast, item 2 (i.e., “I understand that everyone experiences suffering at some point in their lives”), item 7 (i.e., “I understand that feeling upset at times is part of human nature”), item 12 (i.e., “Like me, I know that other people also experience struggles in life”), and item 17 (i.e., “I know that we can all feel distressed when things do not go well in our lives”) exhibit lower discrimination and are less useful in evaluating the latent trait. These four items are associated with understanding the universality of suffering. According to these findings, the items related to AM were able to differentiate employees based on their levels of self-compassion, while the items related to US were not. According to Strauss et al. (2016) review, the definition of compassion often includes related terms such as empathy, which are used to define each other. It is illuminating to consider the overlap and differences between these terms. One of the key distinctions between compassion and empathy is that acting or the desire to act to alleviate suffering is viewed as a core feature of compassion, but not empathy. The Total Information Curve (TIC) supports the overall effectiveness of the SOCS-S, demonstrating precise estimation of scores across an extensive range of the continuum with a low standard error of estimation (SEE).

Theoretical definitions of compassion describe it as a cognitive, affective, and behavioral process consisting of five elements: (a) recognizing suffering, (b) understanding that suffering is a part of every human experience, (c) feeling empathy and relating to those who suffer, (d) tolerating unpleasant feelings caused by suffering, and (e) taking action to ease discomfort (Strauss et al., 2016). Nonetheless, the theory of self-compassion attempts to operationalize a multidimensional description by consolidating various conceptualizations. Our findings suggest an insufficient balance of item difficulty across distinct domains of self-compassion operationalized by the SOCS-S. Seven items (Item 1, 2, 6, 7, 12, 16, 17) had values of *b*_1_ (corresponding to the lowest response category) below −3. Additionally, the parameters *b*1 to *b*3 were predominantly negative, indicating that individuals low on self-compassion tended to respond to SOCS-S items with high response categories (3–5) suggesting that the items are formulated too “easy.” Moreover, all SOCS-S items employ negative phrasing, such as sadness, fear, anger, frustration, guilt, shame, etc., which may also contribute to social desirability biases. Negative phrasing alone may not be sufficient to create difficulty in selecting items that reflect self-compassion, as items that express other socially desirable personality traits, such as self-esteem, self-pity, self-forgiveness, empathy, etc., may also be well-received.

Network analysis showed that item 20 (i.e., “When I’m upset, I do my best to take care of myself”) and item 10 (i.e., “When I’m going through a difficult time, I try to look after myself”) had the relatively higher indicators of centrality: strength, closeness, and betweenness, while item 1 (i.e., “I’m good at recognizing when I’m feeling distressed”) had the lowest centrality indicator values. Therefore, the results acquired in the network analysis are comparable to those discovered in the GRM study. Moreover, the items that scored the highest on indices of centrality in network analysis also showed themselves to be highly informative for the latent construct, as was brought to light by GRM.

The contributions of this study are threefold. First, this is the very first validation study of the SOCS-S conducted among employed individuals in China. Previous validation studies using modified versions of the SOCS-S were conducted in Western countries (de Krijger et al., 2022; Lucarini et al., 2022) and Asian cultures (Kim and Seo, 2021). Second, classical tests and modern methods were used to calculate the reliability. More specifically, the McDonald’s ω coefficient and Cronbach’s α were computed in CTT. The graphs of IIF and TIF marginal reliability were calculated using a modern methodology. While the reliability of the CTT only shows the mean value of all items, IRT has the benefit of offering a bottom-up view of reliability through the IIF graph. More precisely, it records different changes according to the pattern of each item. Hence, this study offers a more practical approach to the psychometric qualities of the SOCS-S. Third, this is the first study to apply network analysis experimental method to the SOCS-S. By employing IRT and network analysis, we were able to identify the most difficult, discriminant, and central items on the SOCS-S.

## Limitations and future directions

There are certain limitations to the generalizability of these findings. First, the use of self-reported measures introduces potential biases such as social desirability and recall biases, highlighting the need for further longitudinal studies or the use of non-self-report measures to triangulate the SOCS-S results, which would be useful for future studies. Second, while self-compassion has been studied as an underlying trait that affects individuals’ perceptions and outcomes in organizations, recent attention in management suggests that practicing self-compassion can lead to happier, more successful, and resilient employees (Chen, 2018). Therefore, future research should investigate the relationships between the aforementioned constructs, including the antecedents and outcomes of self-compassion, using the SOCS-S. Third, research in psychology, pedagogy, and counseling suggests that self-compassion can uniquely contribute to positive responses to challenges and is a source of comfort (Neff, 2003; Neff et al., 2007; Neff and Vonk, 2009; Ma et al., 2022). Therefore, future studies could explore the positive impact of self-compassion across various industries to deepen our understanding. Fourth, the SOCS-S requires further validation as some psychometric properties, such as test–retest reliability, and convergent and discriminant validity, were not assessed in this study due to its scope.

## Conclusion

In general, the evidence from this study utilizing advanced psychometric testing (e.g., IRT and network analysis) confirmed that the SOCS-S possesses strong homogeneity, discriminative power, and excellent reliability. As a result, the SOCS-S is a robust measurement tool that can be effectively employed in both practical and research settings to assess levels of self-compassion among individuals in the Chinese workplace.

## Data availability statement

The raw data supporting the conclusions of this article will be made available by the authors, without undue reservation.

## Ethics statement

The studies involving human participants were reviewed and approved by the Local Ethics Committee at Tianjin Normal University. The patients/participants provided their written informed consent to participate in this study.

## Author contributions

HM and XW conceptualized the manuscript, drafted the manuscript, and conducted all the statistical analyses. HL revised the manuscript. HM collected the data. All authors contributed to the article and approved the submitted version.

## Conflict of interest

The authors declare that the research was conducted in the absence of any commercial or financial relationships that could be construed as a potential conflict of interest.

## Publisher’s note

All claims expressed in this article are solely those of the authors and do not necessarily represent those of their affiliated organizations, or those of the publisher, the editors and the reviewers. Any product that may be evaluated in this article, or claim that may be made by its manufacturer, is not guaranteed or endorsed by the publisher.

## Supplementary material

The Supplementary material for this article can be found online at: https://www.frontiersin.org/articles/10.3389/fpsyg.2023.1110076/full#supplementary-material
