# Supplementary material for: The Sussex Oxford Compassion for the Self Scale validity in a working sample using classical test theory, item response theory and network analysis
Source: Front Psychol. 2023 May 12;14:1110076. doi: 10.3389/fpsyg.2023.1110076 (PMC10213251; doi:10.3389/fpsyg.2023.1110076)
Supplement: Supplementary file 1 [file Table_1.docx]

***Supplementary Materials***

| **Supplementary Table1: The Chinese version of the SOCS-S** | | | | | | |
| --- | --- | --- | --- | --- | --- | --- |
| *请为每个条目选择合适的一项。 | | | | | | |
|  | 条目 | 完全不符合 | 基本不符合 | 不确定 | 基本符合 | 完全符合 |
| 1 | 我很善于识别我痛苦时的感受 |  |  |  |  |  |
| 2 | 我知道每个人都会在生命中的某些阶段经历痛苦 |  |  |  |  |  |
| 3 | 当我经历困难时，我会善待自己 |  |  |  |  |  |
| 4 | 当我情绪低落时，我试着敞开心扉接受自己的感受，而不是回避它们 |  |  |  |  |  |
| 5 | 当我苦恼时，我尽量让自己感觉好一点，即使我对此无能为力 |  |  |  |  |  |
| 6 | 我会注意到我正在经历的痛苦 |  |  |  |  |  |
| 7 | 我明白偶尔感到不安是人生常态 |  |  |  |  |  |
| 8 | 当我遭遇不幸时，我会关心自己 |  |  |  |  |  |
| 9 | 我与自己的痛苦持联结，而不是让它压倒我。 |  |  |  |  |  |
| 10 | 遇到困难时，我会尽量照顾好自己 |  |  |  |  |  |
| 11 | 我很快就注意到自己痛苦的早期迹象 |  |  |  |  |  |
| 12 | 我知道其他人也像我一样在生活中会经历挣扎 |  |  |  |  |  |
| 13 | 当我心烦意乱时，我试着调整我的感受 |  |  |  |  |  |
| 14 | 我与自己的痛苦保持联结，而不是批判自己 |  |  |  |  |  |
| 15 | 当我感到不安时，我尽量做对自己最有利的事 |  |  |  |  |  |
| 16 | 我能识别自己的痛苦迹象 |  |  |  |  |  |
| 17 | 我知道当生活中的事情进展不顺利时，我们都会感到痛苦 |  |  |  |  |  |
| 18 | 即使我对自己很失望，我也能在困境中善待自己 |  |  |  |  |  |
| 19 | 当我心烦意乱时，我会让情绪顺其自然而不受它控制 |  |  |  |  |  |
| 20 | 当情绪低落时，我尽力照顾好自己 |  |  |  |  |  |

| **Supplementary Table 2** Pearson correlations between SOCS**-S** and subscales. | | | | | | |
| --- | --- | --- | --- | --- | --- | --- |
|  | RS | US | FS | TF | AM | Tol |
| RS | — |  |  |  |  |  |
| US | 0.631^**^ | — |  |  |  |  |
| FS | 0.557^**^ | 0.561^**^ | — |  |  |  |
| TF | 0.574^**^ | 0.504^**^ | 0.789^**^ | — |  |  |
| AM | 0.544^**^ | 0.531^**^ | 0.846^**^ | 0.781^**^ | — |  |
| Tol | 0.779^**^ | 0.754^**^ | 0.899^**^ | 0.878^**^ | 0.888^**^ | — |
| **p* < .05, ***p* < .01. | | | | | | |


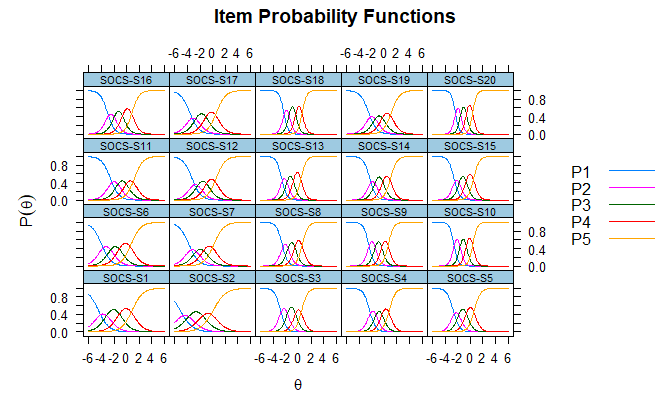


**Figure S1. Item Characteristic Curve**
